# Unmet Need for Completing the Antenatal Care Continuum Among Pregnant Women in Rural India: A Mixed-Methods Study

**DOI:** 10.7759/cureus.87524

**Published:** 2025-07-08

**Authors:** Venkatashiva Reddy B, Vishnu Rajan, Rajeev Aravindakshan, Suresh Vaikkakara, Vijayan Sharmila

**Affiliations:** 1 Department of Community and Family Medicine, All India Institute of Medical Sciences, Mangalagiri, Mangalagiri, IND; 2 Department of Endocrinology, All India Institute of Medical Sciences, Mangalagiri, Mangalagiri, IND; 3 Department of Obstetrics and Gynecology, All India Institute of Medical Sciences, Mangalagiri, Mangalagiri, IND

**Keywords:** antenatal care, high-risk pregnancy, maternal health services, primary health care services, thyroid diseases

## Abstract

Introduction

India’s National Health Programs aim to provide quality antenatal care (ANC). However, gaps remain, particularly in the availability of essential diagnostics such as thyroid function tests, which are critical for identifying high-risk pregnancies (HRPs). This study explores the proportion of missed at-risk pregnancies due to undiagnosed thyroid disorders and the associated out-of-pocket expenditure (OOPE) in rural Andhra Pradesh.

Methodology

A convergent parallel mixed-methods design was adopted, combining a cross-sectional study with focus group discussions and in-depth interviews. Quantitative data were collected from 96 pregnant women attending primary health centers (PHCs). Qualitative insights were gathered from healthcare providers and pregnant women with missed thyroid disorder diagnoses. The study was conducted over a one-year period (2024-2025) across four PHCs in Mangalagiri Mandal, Andhra Pradesh, India. Blood samples for thyroid function testing were collected at the PHCs and transported to a central laboratory at a tertiary care facility using drone technology.

Results

The study included women with a mean age of 24.0 years, most of whom were housewives with at least a high school education. Among them, 59% had not undergone prior thyroid testing, and 5% were found to have undiagnosed hypothyroidism. Women without prior testing were more likely to be in their first trimester and classified as HRPs. Most diagnostic investigations were performed at private facilities, with thyroid-stimulating hormone testing costing a median of ₹500.

Conclusions

While the National Health Program has strengthened ANC, critical gaps remain, particularly in the early diagnosis of thyroid disorders at the primary care level. Strengthening diagnostic capabilities, reducing OOPE, and ensuring timely follow-up for HRPs are essential to lowering maternal and neonatal mortality in rural settings.

## Introduction

The Pradhan Mantri Surakshit Matritva Abhiyan (PMSMA) in India offers fixed-day quality antenatal care (ANC) to pregnant women on the ninth of each month [1]. The initiative aims to ensure free, assured, and comprehensive antenatal services are delivered at designated health facilities by obstetricians and medical officers. Since its inception in 2016, PMSMA has been implemented across more than 20,000 healthcare facilities and has provided antenatal check-ups to approximately 47 million pregnant women nationwide [2]. According to existing literature, 20-30% of pregnancies are classified as high risk, accounting for nearly 75% of perinatal morbidity and mortality in India [3,4]. However, only 14% of pregnancies are currently being reported as “high risk” on the PMSMA platform, with significant variation across states [5]. With an estimated 30,000 maternal deaths annually, India’s high maternal mortality ratio remains a major concern. It is therefore critical to provide quality ANC to every pregnant woman, accurately identify high-risk pregnancies (HRPs), and ensure they are tracked for counseling, management, birth preparedness, and referral until delivery to close the care loop.

Despite its broad reach, PMSMA continues to face key challenges, particularly the lack of essential laboratory investigations such as thyroid function tests. Thyroid dysfunction during pregnancy is a known contributor to adverse maternal and neonatal outcomes, including preterm birth, preeclampsia, fetal growth restriction, and neurocognitive impairment in infants [6]. Studies report that subclinical and overt hypothyroidism affect approximately 4-10% of pregnant women, highlighting the need for timely detection and intervention [7]. A meta-analysis of observational studies estimated the prevalence of hypothyroidism among pregnant women in India at 11.07%, with subclinical hypothyroidism more common than overt hypothyroidism [8]. Although thyroid function tests are included in PMSMA’s onsite monitoring checklists, many primary health centers (PHCs) lack the resources and infrastructure to perform these investigations. Consequently, numerous pregnant women either go undiagnosed or are forced to seek diagnostic services at private facilities, leading to delayed care, increased out-of-pocket expenditure (OOPE), and heightened health risks. Laboratory tests account for a significant portion of the total OOPE during pregnancy in India, compounding the financial burden [9,10]. In addition, geographic and infrastructural barriers, particularly in rural and remote regions, further limit access to necessary diagnostics and care.

Innovative technologies such as drone-based delivery systems offer promising solutions to bridge these diagnostic access gaps in underserved areas. Drones can be utilized to transport laboratory samples from sub-centers or PHCs to better-equipped facilities and return with diagnostic results or medications, thereby significantly reducing turnaround time. Pilot initiatives like the “Medicine from the Sky” project in Telangana and Arunachal Pradesh have demonstrated the potential of drone technology in improving healthcare delivery in remote locations [11]. This approach circumvents the logistical and financial challenges of establishing diagnostic infrastructure, such as thyroid testing, at the primary care level, making advanced diagnostics more accessible without extensive investment in local facilities.

This study was designed to explore unmet needs in maternal health services under India’s National Health Programs. Its primary objective was to determine the proportion of missed at-risk pregnancies due to undiagnosed thyroid disorders at primary healthcare centers in rural Andhra Pradesh. The study also examined OOPE for antenatal services, preferred healthcare facilities, and the reasons guiding these preferences. Although the direct use of drones was not evaluated, their application for transporting diagnostic samples from PHCs to tertiary care centers was considered as a potential intervention.

## Materials and methods

Study design

This study adopted a convergent parallel mixed-methods design, integrating both quantitative and qualitative approaches to evaluate the proportion of missed risk assessments due to the unavailability of essential laboratory tests at the primary healthcare level and the subsequent impact on referral and management of maternal health conditions during pregnancy. The quantitative component consisted of a cross-sectional study, while the qualitative component included focus group discussions (FGDs) and in-depth interviews (IDIs) with accredited social health activists (ASHAs), auxiliary nurse midwives (ANMs), and pregnant women from the field practice area.

Study setting, sample size, and sampling

The study was conducted over a one-year period (2024-2025) across four PHCs located in Mangalagiri Mandal, Andhra Pradesh, India. The Centre for Rural Health at All India Institute of Medical Sciences (AIIMS), PHC Nutakkhi, served as the primary field practice area with a catchment population of approximately 10,000. Based on the Guntur district birth rate of 14.7 per 1,000 population, an estimated 147 pregnancies occur annually within this population. According to the Rapid Survey on Children 2013-14, 65.7% of pregnant women in the region accessed ANC services through government facilities [12]. Using this data, a sample size of 96 was calculated, assuming 80% power and a 5% alpha error. Consecutive sampling was used to enroll eligible pregnant women attending the selected primary healthcare centers during the study period. Participants were included after providing written informed consent, following a review of the participant information sheet.

For the qualitative component, FGDs were conducted with groups of seven to eight primary healthcare providers, while IDIs were held with five pregnant women who had experienced missed diagnoses during their antenatal visits. Ethical approval for the study was obtained from the institute’s ethics committee.

Study participants: inclusion and exclusion criteria

Inclusion criteria included pregnant women aged 19 to 45 years, married, and attending PMSMA days at the selected PHCs during the study period. For the qualitative phase, ASHAs and ANMs from Nutakkhi village were recruited.

Exclusion criteria were applied to pregnant women who had a preexisting diagnosis of thyroid disorders at the time of their visit to the PHC. Additionally, women with known intellectual disabilities or psychiatric illnesses were excluded from participation.

Study procedure

Quantitative Data

For the quantitative component, relevant clinical and demographic information was extracted from the medical records of participating pregnant women. This included age, gestational age, presenting symptoms, antenatal and family history, treatment history, and any documented risk factors for thyroid disorders. Additionally, a semi-structured questionnaire was administered to gather data on OOPE and the type of healthcare facility accessed for antenatal services.

According to the 2017 American Thyroid Association guidelines, hypothyroidism during pregnancy is defined by thyroid-stimulating hormone (TSH) levels exceeding 4 mIU/L. Subclinical hypothyroidism is diagnosed when elevated TSH is accompanied by normal free thyroxine (T4), while overt hypothyroidism involves TSH levels typically greater than 10 mIU/L with low free T4 concentrations [7].

Blood samples were collected from pregnant women attending PMSMA days at the selected PHCs. Following standard venipuncture procedures, approximately 5 mL of blood was drawn into EDTA Vacutainer tubes. Each sample was labeled with a unique identifier to maintain confidentiality and anonymity.

To facilitate prompt thyroid function testing, samples collected at the PHCs were transported to a central laboratory at a tertiary care center - AIIMS Mangalagiri - using drone technology within a cold chain. The drone operations were coordinated by a multidisciplinary team comprising healthcare professionals from AIIMS Mangalagiri, certified drone operators, and field staff. On average, the drone transit time from PHC Nutakkhi to AIIMS Mangalagiri was seven to eight minutes. A designated laboratory technician and a nursing officer conducted preflight and postflight sample verification and documentation. Drone services were deployed weekly for transporting blood samples, including those for complete blood count, glucose profile, lipid profile, and thyroid function testing, from the Centre for Rural Health at PHC Nutakkhi to AIIMS Mangalagiri.

Qualitative Data

The qualitative component comprised FGDs with seven to eight primary healthcare providers - ASHAs and ANMs - at each selected site. These discussions aimed to explore their insights on the challenges posed by the unavailability of essential laboratory tests, particularly in managing maternal health and facilitating timely referrals. Additionally, IDIs were conducted with five randomly selected pregnant women beyond 28 weeks of gestation who had experienced missed diagnoses. The interviews captured their experiences, the barriers they encountered, and their perceptions of the referral process.

Statistical analysis

Quantitative Analysis

Data were collected using the Epicollect5 mobile application and analyzed using IBM SPSS Statistics for Windows, Version 29.0 (Released 2022; IBM Corp., Armonk, NY, USA). Categorical variables were presented as frequencies and percentages, while continuous variables were described using means with standard deviations or medians with IQRs, based on data distribution. Inferential statistics, including chi-square tests and logistic regression, were applied to identify associations between missed risk classification, referral patterns, and demographic variables. A p-value of less than 0.05 was considered statistically significant.

Qualitative Analysis

Audio recordings from FGDs and IDIs were transcribed verbatim within 24 hours of data collection. Transcriptions in the local language were translated into English during the process. A second researcher reviewed the transcripts to minimize bias and enhance interpretive validity. Qualitative data were analyzed thematically using an inductive coding approach in ATLAS.ti version 8.0 (ATLAS.ti Scientific Software Development GmbH, Berlin, Germany). Codes were generated from the data and used to identify emerging themes relevant to the study objectives.

## Results

The study population had a mean age of 24.0 years (SD: 3.3), with the majority, 65 (68%), aged ≤25 years. Most participants were housewives (90; 93.7%), and 33 (34.3%) had attained at least a high school education. Nearly half of the participants (46; 48%) belonged to the upper middle class according to the Modified BG Prasad Socioeconomic Scale [13]. Extended family structures were common, reported by 76 participants (79.1%), and the mean age at marriage was 21.15 years (SD: 2.96). Consanguineous marriages were observed in 15 cases (15.6%). Regarding obstetric history, 39 participants (40.6%) were gravida 2, and 16 (16.6%) had a history of stillbirth. Most women had one living child (41; 42.7%), and 44 (46%) were in their second trimester at the time of the study (Table 1).

**Table 1:** Comparison of characteristics across pregnant women with and without prior testing ^*^ Chi-squared test ^#^ χ² statistic

Characteristics	Pregnant women with previous thyroid tests				Total		p-value^*^	Test statistic^#^
	Yes (n = 39)		No (n = 57)					
	n	%	n	%	n	%		
Age of the pregnant woman								
≤25 years	28	71.79	39	68.42	67	69.79	0.268	2.634
≥26 years	11	28.21	18	31.58	29	30.21		
Occupation of the pregnant woman								
Cooli/daily labor	0	0	1	1.75	1	1.04	0.953	1.591
Housewife/retired	37	94.87	53	92.98	90	93.75		
Government job	0	0	1	1.75	1	1.04		
Private job	2	5.13	2	3.51	4	4.17		
Education of the pregnant woman								
Illiterate	0	0	1	1.75	1	1.04	0.86	6.97
Primary	1	2.56	0	0	1	1.04		
High school	13	33.33	20	35.09	33	34.38		
Middle	0	0	2	3.51	2	2.08		
Intermediate	11	28.21	12	21.05	23	23.96		
Graduate	11	28.21	19	33.33	30	31.25		
Post graduate	3	7.69	3	5.26	6	6.25		
Occupation of the husband								
Cooli/daily labor/others	22	56.41	26	45.61	48	50	0.017	21.65
Farmer	0	0	4	7.02	4	4.17		
Fisheries	1	2.56	0	0	1	1.04		
Government job	1	2.56	2	3.51	3	3.13		
Private job	11	28.21	23	40.35	34	35.42		
Small business	4	10.26	2	3.51	6	6.25		
Education of the husband								
Illiterate	2	5.13	2	3.51	4	4.17	0.793	9.56
Primary	3	7.69	0	0	3	3.13		
High school	3	7.69	4	7.02	7	7.29		
Middle	12	30.77	14	24.56	26	27.08		
Intermediate	4	10.26	7	12.28	11	11.46		
Graduate	11	28.21	25	43.86	36	37.5		
Post graduate	3	7.69	5	8.77	8	8.33		
Socioeconomic status (Modified BG Prasad Scale)								
Lower	2	5.13	0	0	2	2.08	0.801	4.58
Lower middle	1	2.56	1	1.75	2	2.08		
Middle	8	20.51	14	24.56	22	22.92		
Upper middle	19	48.72	27	47.37	46	47.92		
Upper	8	20.51	15	26.32	23	23.96		
Type of the family								
Extended	34	87.18	42	73.68	76	79.17	0.26	2.65
Nuclear	5	12.82	15	26.32	20	20.83		
Single	0	0	0	0	0	0		
Consanguineous marriage								
Yes	7	17.95	8	14.04	15	15.63	0.771	0.52
No	32	82.05	49	85.96	81	84.38		
Type of house								
Kacha/semi-pucca	8	20.51	11	19.3	19	19.79	0.863	0.294
Pucca	30	76.92	46	80.7	76	79.17		
Gravida								
0	2	5.13	5	8.77	7	7.29	0.789	6.305
1	16	41.03	18	31.58	34	35.42		
2	18	46.15	21	36.84	39	40.63		
3	3	7.69	11	19.3	14	14.58		
4	0	0	1	1.75	1	1.04		
5	0	0	1	1.75	1	1.04		
Still birth	6	15.38	10	17.54	16	16.67	0.844	0.338
Parity								
0	13	33.33	16	28.07	29	30.21	0.735	2.004
1	21	53.85	36	63.16	57	59.38		
2	1	2.56	4	7.02	5	5.21		
Live children								
0	15	38.46	19	33.33	34	35.42	0.642	2.513
1	13	33.33	28	49.12	41	42.71		
2	1	2.56	2	3.51	3	3.13		
Abortion								
0	18	46.15	31	54.39	49	51.04	0.796	3.1
1	9	23.08	12	21.05	21	21.88		
2	1	2.56	0	0	1	1.04		
3	0	0	1	1.75	1	1.04		
Gestational age								
First trimester	5	12.82	20	35.09	25	26.04	0.044	9.79
Second trimester	20	51.28	24	42.11	44	45.83		
Third trimester	13	33.33	13	22.81	26	27.08		
High-risk pregnancy	6	15.38	21	36.84	27	28.13	0.999	16.179
Comorbidities present	17	43.59	24	42.11	41	42.71	0.508	1.356

Thyroid testing had been conducted previously in 39 women (41%), while 57 (59%) had not undergone any prior testing. A statistically significant association was observed between the husband’s occupation and prior thyroid testing, with a higher proportion of private-sector jobholders among those who had not been tested (40.3% vs. 28.1%, p = 0.017). Additionally, a greater proportion of women in their first trimester had not undergone thyroid testing (35.1% vs. 12.8%, p = 0.04). Notably, 36.8% of women without prior testing were classified as HRPs, compared to 15.3% among those who had been tested (p = 0.99). No statistically significant differences were observed between the tested and untested groups with respect to age, occupation, education level, socio-economic status, family type, consanguinity, gravida, parity, number of live births, history of stillbirths, abortions, or comorbidities (Table 1).

Table 2 presents the biochemical parameters of pregnant women who had not undergone prior thyroid testing. The mean total triiodothyronine (T3) level was 197.86 ng/dL (SD: 48.61), the mean total thyroxine (T4) level was 12.07 µg/dL (SD: 2.53), and the mean TSH level was 2.0 µIU/mL (SD: 1.01).

**Table 2:** Biochemical parameters among pregnant women T3, triiodothyronine; T4, thyroxine; TSH, thyroid-stimulating hormone

S. no.	Biochemical parameters	n (%)	Mean	SD	IQR 1	Median	IQR 3	IQR
1	Total T3	56 (58.3)	197.8	48.6	162.25	194.5	227.75	65.5
2	Total T4	56 (58.3)	12.07	2.5	10	12	13.75	3.75
3	TSH (mIU/L)	56 (58.3)	2	1	1	2	2	1
4	Hypothyroidism							
	Yes (>4 IU/ml), n (%)	5 (5.3)						
	No (<4 IU/ml), n (%)	90 (95)						
5	Hemoglobin (g/dl)	33 (34.3)	9.7	1.27	9.2	9.9	10.8	1.6

The median body weight of the participants was 57 kg (IQR: 50.25-66.5), and the median height was 155.2 cm (IQR: 152-159). The median pulse rate was 92 beats per minute (IQR: 80-100), while the median respiratory rate was 18 breaths per minute (IQR: 16-20). Median resting systolic and diastolic blood pressures were 103 mmHg (IQR: 98.25-114.25) and 69.5 mmHg (IQR: 61-74), respectively (Table 3).

**Table 3:** General physical examination among pregnant women

S. no.	Biochemical parameters	n (%)	Mean	SD	IQR 1	Median	IQR 3	IQR
1	Body weight (kg)	88 (91.67)	59.6	13	50.25	57	66.5	16.25
2	Height (cm)	86 (89.58)	155.6	5.9	152	155.2	159	7
3	Body temperature (F)	41 (42.70)	85.1	25.1	94.5	97.6	98.5	4
4	Pulse rate (beats/min)	63 (65.62)	90.1	13.4	80	92	100	20
5	Respiratory rate (cycles/min)	44 (45.83)	20.4	13.4	16	18	20	4
6	Systolic blood pressure (mmHg)	82 (85.41)	104.7	12.2	98.25	103	114.25	16
7	Diastolic blood pressure (mmHg)	82 (85.41)	68.3	10.4	61	69.5	74	13
8	Hemoglobin (g/dl)	33 (34.37)	9.7	1.27	9.2	9.9	10.8	1.6

A total of 59 participants (61.4%) underwent ultrasonography (USG) of the abdomen and pelvis, 37 (38.5%) had TSH testing, 43 (44.8%) underwent random blood sugar (RBS) testing, and 39 (40.6%) were tested for urine glucose. Additionally, 12 participants (12.5%) underwent an oral glucose tolerance test (OGTT), and 54 (56.2%) were screened for syphilis. Most of these investigations, 47 (79.6%), were conducted in private hospitals, with the exception of urine glucose and syphilis testing, which were primarily performed in government facilities. The median cost of investigations varied, with USG being the most expensive at ₹1000 (IQR: ₹1000), and RBS the least expensive at ₹50 (IQR: ₹70). The median cost of TSH testing was ₹500 (IQR: ₹434.5). Most tests were performed once, except for USG, which had a median frequency of two (IQR: 2) and a maximum of six times (Table 4).

**Table 4:** Details of antenatal blood investigations by pregnant women who had prior testing OGTT, oral glucose tolerance test; RBS, random blood sugar; TSH, thyroid-stimulating hormone; USG, ultrasonography

Investigation	USG abdomen and pelvis	TSH	RBS	Urine glucose	OGTT	Syphilis
Investigations done, n (%)	59 (61.45)	37 (38.54)	43 (44.79)	39 (40.62)	12 (12.5)	54 (56.25)
Place of investigations						
Government hospital, n (%)	12 (20.34)	0	14 (32.55)	30 (76.92)	8 (66.67)	37 (68.51)
Private hospital, n (%)	47 (79.66)	37 (100)	29 (67.45)	9 (23.08)	4 (33.33)	17 (31.49)
Cost of investigation (INR)						
n (%)	53 (55.20)	23 (23.90)	35 (36.45)	39 (40.62)	12 (12.50)	44 (45.83)
Median	1000	500	50	150	150	200
IQR	1000	434.5	70	180	375	400
Minimum value	200	150	0	0	0	0
Maximum value	4500	960	100	180	500	800
Number of times the investigation was done						
n (%)	40 (41.61)	23 (23.90)	33 (34.3)	36 (37.5)	12 (12.50)	39 (40.62)
Median	2	1	1	1	1	1
IQR	2	0	0	0	0	0
Minimum value	1	1	1	1	1	1
Maximum value	6	8	10	10	1	10

The findings from the thematic analysis of the IDIs and FGDs are presented in Table 5 and Table 6, respectively.

**Table 5:** Thematic analysis of in-depth interviews with pregnant women

Objective	Theme	Code	Quotation
I	Importance of early detection	Regular testing	“We don’t neglect regular scanning, and blood tests are compulsory.”
	Awareness of pregnancy complications	Knowledge of pregnancy-related illnesses	“Yes, I know. I already studied that.”
	Timing of tests	Timing for testing	“I think we need to take tests in the second month ending only.”
II	Timeliness and availability of care	Delayed response	“PHC, they came very late. What if something happens to the baby?”
	Availability of essential tests	Essential tests conducted	“All the blood tests and urine tests, and when you have thyroid, that test also.”
	Suggestions for service improvement	Need for emergency services	“No one is available in the evening in case of any emergency labor pain.”
III	Seeking private healthcare	Referral to private hospitals	“My doctor from the holistic hospital referred me to rainbow hospital.”
	Financial burden	Cost concerns	Inferred from the context of seeking private care (not directly quoted)
IV	Supportive healthcare workers	Support from ASHA workers	“The ASHA worker used to take me to the government hospital every week.”
	Attendance at maternal programs	Participation in PMSMA	“Yes, every month on the ninth.”

**Table 6:** Thematic analysis of focused group discussions with primary healthcare providers

Theme	Code	Quotation
Tests required during pregnancy	Regular monitoring	“We will check regular height and weight throughout the pregnancy.”
	Essential lab tests	“Usual tests are glucose, thyroid, and the VDLR test.”
Out-of-pocket expenditure	Cost of tests	“Easily Rs. 2000 to 3000.”
	Scanning costs	“Each time they will spend around Rs. 1500-3000.”
Referral practices	Referral to government hospitals	“Yes, we will refer to the nearby government hospital.”
	Re-testing in private hospitals	“Even if they tested here again in a private hospital, they do all the tests for money.”
Availability of tests at PHC	Tests available	“Urine tests, glucose test.”
	Missing tests	“Even in Tenali, also, thyroid tests were not available. Only scanning and remaining tests are free.”
Test accessibility	Timing of OGTT tests	“But they want it early in the morning only, ma’am; here it is taking too long.”

## Discussion

The findings of this study underscore a critical gap in the national health program for maternal care at the primary healthcare level, particularly in the identification and management of HRPs due to the unavailability of essential laboratory testing. Although the program aims to provide assured and comprehensive ANC, the absence of thyroid function tests in many PHCs remains a significant obstacle to achieving optimal maternal and neonatal health outcomes. This study further offers insight into how pregnant women and frontline health workers perceive this gap, their healthcare-seeking preferences, and the challenges they encounter in accessing essential blood investigations.

The majority of pregnant participants were young, with 68% aged 25 years or younger, and most were multigravida. Notably, 57 women had not undergone thyroid testing during the current pregnancy. Among these, 5% were found to have undiagnosed hypothyroidism, suggesting missed opportunities for early detection. This is consistent with previous findings indicating that subclinical and overt hypothyroidism during pregnancy in India have a prevalence of 6.47-9% and 3-4.58%, respectively [14]. A notable association was observed between prior thyroid testing and the occupation of the husband, particularly among those working as coolies or daily wage laborers. This suggests a possible link between economic status and access to healthcare, as financial resources may influence both the ability and willingness to pursue blood investigations [15].

Gestational age was significantly different between those who had undergone thyroid testing and those who had not, with a higher proportion of tested women being in the second trimester. This may reflect increased ANC visits and physician-recommended tests during this critical period [16]. Among the untested women, over one-third were classified as HRPs, thereby elevating the risk of adverse maternal and neonatal outcomes. This aligns with national data from PMSMA days in India, where the prevalence of HRPs is estimated to be around 40% [3]. Anemia emerged as the most common cause of HRP in India [17,18], and in this study, the mean hemoglobin levels indicated a moderate severity of anemia among participants. Given the high burden of anemia among Indian pregnant women [19,20], targeted strategies such as iron supplementation and dietary modifications remain essential.

Biochemical assessments revealed that average total T3 and T4 levels were within normal reference ranges, while the mean TSH level was 2 mIU/L, suggesting that most participants were euthyroid. Nonetheless, the potential presence of subclinical hypothyroidism remains a concern [21].

The study also examined the location and cost of ANC-related blood investigations. A significant number of women had their tests done in private healthcare facilities, especially TSH testing, with 38 women undergoing this test privately. The median cost for TSH testing was INR 500, which could be financially burdensome for low-income households [22]. The median OOPE during the antenatal period in India stands at INR 5,000, with diagnostic investigations constituting the highest share [23]. The lack of thyroid testing in the PMSMA program may therefore impose an additional economic strain on pregnant women.

Thematic analysis of FGDs and IDIs revealed key determinants of maternal healthcare-seeking behavior. Pregnant women acknowledged the importance of early diagnosis and regular antenatal testing, consistent with findings from previous maternal health studies [24,25]. However, many reported the need to visit multiple facilities to obtain comprehensive care, often constrained by financial and logistical challenges. Healthcare workers, including ASHAs and ANMs, voiced concerns about the unavailability of diagnostic services at PHCs, which hinders timely referrals and continuity of care. These insights echo the broader literature emphasizing the necessity of integrated and patient-centered ANC services [26,27].

Despite the wide reach of the PMSMA program, a persistent unmet need remains, especially for onsite thyroid function testing at PHCs. One reason for this gap is the high cost of diagnostic infrastructure, making it impractical for PHCs to independently offer comprehensive testing. An innovative and promising solution is the use of drones to transport blood samples from PHCs to centralized laboratories for thyroid function testing. This approach can help bypass infrastructural and geographical limitations, reduce turnaround times, and enable timely detection and management of thyroid disorders in pregnant women, even in resource-constrained settings [28].

A notable strength of this study is its mixed-methods design, which combines quantitative data with qualitative insights from pregnant women and healthcare providers. This approach provided a nuanced understanding of the gaps in maternal health services, particularly the lack of thyroid function testing, and its implications for maternal and neonatal outcomes. The study also introduces the potential of drone-based sample transportation as a practical and forward-thinking strategy to address diagnostic gaps. Furthermore, it highlights the significant financial barriers posed by OOPE. However, the study is limited by its geographically restricted setting and potential recall bias in self-reported expenditure data. The absence of long-term follow-up also limits conclusions about clinical outcomes over time.

## Conclusions

While the national health program has made significant progress in expanding ANC coverage, critical gaps remain in delivering comprehensive ANC at the primary care level, particularly in the timely diagnosis of thyroid disorders. Addressing these gaps through improved diagnostic access, financial protection, and systematic tracking of HRPs is vital for achieving program goals related to reducing maternal and neonatal mortality. Integrating drone-based sample transport offers a promising, scalable solution to overcome logistical challenges and enhance the diagnostic capacity of primary healthcare centers, particularly in rural and underserved regions.
